# Supplementary material for: Examination of multilevel domains of minority stress: Implications for drug use and mental and physical health among Latina women who have sex with women and men
Source: PLoS One. 2020 Mar 26;15(3):e0230437. doi: 10.1371/journal.pone.0230437 (PMC7098621; doi:10.1371/journal.pone.0230437)
Supplement: S1 Appendix — (DOCX) [file pone.0230437.s001.docx]

| **Appendix. Missing on Sexual Identity** | | | | |
| --- | --- | --- | --- | --- |
|  | No (%) | Yes (%) | x2 | p |
| Age (mean/t) | 33.59 | 32.66 | 2.84 | .005 |
| Years of Education (mean/t) | 10.91 | 10.99 | 0.28 | .779 |
| Unemployed/Occasional Employment | 34.2 | 35.4 | 0.03 | .853 |
| Marital Status |  |  | 3.53 | .175 |
| Single, Never Married | 35.8 | 38.0 |  |  |
| Separated/Divorced/Widowed | 11.7 | 20.3 |  |  |
| Married/Cohabitating | 52.5 | 41.8 |  |  |
| Number of Children (mean/t) | 3.49 | 3.16 | 1.28 | .201 |
| Unstably Housed | 20.8 | 21.5 | 0.01 | .908 |
| Ever Incarcerated | 63.0 | 62.8 | 0.00 | .977 |
| Ever had a Same Sex Partner | 31.6 | 28.6 | 0.20 | .651 |
