# Supplementary material for: Examination of multilevel domains of minority stress: Implications for drug use and mental and physical health among Latina women who have sex with women and men
Source: PLoS One. 2020 Mar 26;15(3):e0230437. doi: 10.1371/journal.pone.0230437 (PMC7098621; doi:10.1371/journal.pone.0230437)
Supplement: Attachment — Submitted filename: Response to Reviewers.docx [file pone.0230437.s002.docx]

Please use the space provided to explain your answers to the questions above. You may also include additional comments for the author, including concerns about dual publication, research ethics, or publication ethics. (Please upload your review as an attachment if it exceeds 20,000 characters)

**Reviewer #1**: It seems understandable the incomplete availability of data, as this work is part of a larger ongoing project.

*We have a no-cost extension for the study so technically it has not ended.. Per NIH policy and PLOS ONE’s data policy, the data that support the findings will be made openly available but is not yet ready to be released.*

All that was left was to better conceptualize, at the beginning of the work, what would be WSWM?, that is, how many episodes of sex with women would count to be classified as WSWM?

*For participants in our study, being labeled a “WSWM” is based on lifetime reports (so having sex with one man and one women at least one time). We believed that this is well clarified in the methods section but have made it more explicit in the final paragraph of the introduction.*

As small details:
1. the title is not clear enough

*Reviewer 2 made a similar comment. Please see our response below.*

2. reference 5 has some errors

*Thanks for noticing this. We added the sponsor agency of the report to the citation (National Health Statistics Reports, Centers for Disease Control and Prevention).*

3. DSM IV reference missing

*The reference has been added to the citation list and the in-text citation has been updated.*

I would like to congratulate the authors, it is an interesting and important work. Both, the structure of the article, including the technical aspects, as well as the content are very good.

*Thank you for these kind words. We are excited to share these findings!*

**Reviewer #2**: This is a very interesting and needed article that calls for attention to several key factors that are associated to drug use, physical health and mental health among a sexual minority population - in this case, women who have sex with women and men (WSWM).

Despite being a very specific and vulnerable population, the study results are very interesting and shed light for a poorly understood question. In my opinion, the article could be easily improved if the points listed below are answered/tackled:

1. Would it be possible to adjust the title, possibly making it more attractive? The data shown cover so many different aspects that should not to be simplified as just the "documentation of disparities". Moreover, the socioeconomic vulnerability to which they are exposed (e.g., violence and incarceration) should be another point to be highlighted, instead of just being the context of "living in a disadvantaged community".

*Both Reviewers 1 and 2 commented on the title. Following Reviewer 2’s suggestion, we have simplified it to the following: “*Documentation of Disparities for Women Who Have Sex With Women and Men Using a Cohort of Mexican-American Women.”

2. Include the explanation for not assessing data of women who have sex with women/lesbians in the abstract. It is actually a little bit confusing in the article itself (last paragraph of page 6 and first one of page 7) - since 3.8% answered about their sexual identity as being lesbians, it would not be expected that absolutely 'all women in the cohort reported having sex with men'. Would it be specific of such a population? Moreover, this issue ends up getting more complicated when the results for the sexual identification are observed (lines 251-257) - could you please try to simplify or improve the intelligibility of these excerpts?

*We agree that this is very complex. In our sample, it is correct that all women reported having sex with men independent of their sexual identity. So all of the women in our sample that are lesbian reported having sex with a man at least once. We do not know from our data the context in which these sexual encounters took place: did it occur during sex work? out of social pressure to “fit in”? is their sexual identity fluid? This context is very important and it is missing from our data. The same is true for women who report being heterosexual yet have had sex with women. We were recently awarded a pilot grant to follow up with the WSWM subsample and ask them these kinds of questions.*

*For the current paper, we see identity and behavior as separate, yet related, factors that may contribute to minority stress and negative health outcomes in different ways (similar to studies of the health of men who have sex with men (MSM) regardless of their sexual identity). We have added clarifying statements beginning in the Measures: Individual-level section to make this clearer for the reader.*

3. Please verify the name for 'substance dependence severity scale (SDS)' vs. 'Severity of Dependence Scale (SDS)' (line 188). It would be interesting if authors could explain the reason why they used more conservative cut-off scores as suggested by other researchers - e.g., were the populations similar to the one assessed?

*We fixed the error with the scale title in the text and made some edits to the description of the measure. The scale as originally developed by the WHO uses the same items and cut-off scores for all types of drugs (e.g., cocaine, heroin, meth, marijuana, etc.). Other researchers have developed drug-specific cut-off scores by examining the scale properties specifically for meth users, heroin users, etc. We chose to use these more refined scores in order to be more precise and cautious in our analysis.*

4. In the discussion section, since it is a very particular population, it would be nice to have national rates of the individual-level, interpersonal-level, structural-level and health outcomes that are assessed, or at least identifying those that are rather different from the national level and actually citing the literature rate. For example, line 328 of the article states that the reported rates of childhood trauma, IPV, and incarceration history, overall, far exceed national rates - which are.... I believe this would further show the importance of the present findings and also of how relevant it is to study minority populations like these.

*This is a great suggestion and we have done this.*
