# Supplementary material for: Examination of multilevel domains of minority stress: Implications for drug use and mental and physical health among Latina women who have sex with women and men
Source: PLoS One. 2020 Mar 26;15(3):e0230437. doi: 10.1371/journal.pone.0230437 (PMC7098621; doi:10.1371/journal.pone.0230437)
Supplement: Attachment — Submitted filename: Repsonse to Reviewers.docx [file pone.0230437.s003.docx]

*Additional Editor Comments (if provided):

Dear authors, I will be glad to accept your submission for publication after some small changes suggested by one the reviewers:

"The authors have addressed all comments and the article has improved, but they have misunderstood my first comment regarding the title. While I wrote "The data shown cover so many different aspects that should NOT to be simplified as just the "documentation of disparities", they understood as if I was suggesting to actually use the terms "documentation of disparities". Besides this minor correction and provided a new title that encompasses the range of multi-level risk factors that were assessed in the present study".

Best wishes, Geilson.*

I see how this was a misunderstanding and am glad to have the opportunity to revise the title. The new title is, “Examination of Multilevel Domains of Minority Stress: Implications for Drug Use and Mental and Physical Health among Latina Women who have Sex with Women and Men.”
